# Ethnic differences in the relationship between step cadence and physical function in older adults

**DOI:** 10.1080/02640414.2022.2057013

**Published:** 2022-04-01

**Authors:** Philip McBride, Thomas Yates, Joseph Henson, Melanie Davies, Jason Gill, Carlos Celis-Morales, Kamlesh Khunti, Benjamin Maylor, Alex Rowlands, Charlotte Edwardson

**Affiliations:** a Diabetes Research Centre, Health Sciences, College of Life Sciences, University of Leicester, Leicester, UK; b NIHR Leicester Biomedical Research Centre, University of Leicester and University Hospitals of Leicester HHS Trust, Leicester, UK; c Leicester Diabetes Centre, Leicester General Hospital, University Hospitals Leicester, Leicester, UK; d Institute of Cardiovascular and Medical Sciences, University of Glasgow, Glasgow, UK; e NIHR Collaboration for Leadership in Applied Health Research and Care East Midlands, University of Leicester and University Hospitals of Leicester Nhs Trust, Leicester, UK

**Keywords:** Physical activity, frailty, gait, walking, South Asian

## Abstract

This study investigated associations between step cadence and physical function in healthy South Asian (SA) and White European (WE) older adults, aged ≥60. Participants completed the 60-s Sit-to-Stand (STS-60) test of physical function. Free-living stepping was measured using the activPAL3™. Seventy-one WEs (age = 72 ± 5, 53% male) and 33 SAs (age = 71 ± 5, 55% male) were included. WEs scored higher than SAs in the STS-60 (23 vs 20 repetitions, *p* = 0.045). Compared to WEs, SAs had significantly lower total and brisk (≥100 steps/min) steps (total: 8971 vs 7780 steps/day, *p* = 0.041; brisk: 5515 vs 3723 steps/day, *p* = 0.001). In WEs, 1000 brisk steps and each decile higher proportion of steps spent brisk stepping were associated with STS-60 (*β* = 0.72 95% CI 0.05, 1.38 and *β* = 1.01 95% CI 0.19, 1.82, respectively), with associations persisting across mean peak 1 min (*β* = 1.42 95% CI 0.12, 2.71), 30 min (*β* = 1.71 95% CI 0.22, 3.20), and 60 min (*β* = 2.16 95% CI 0.62, 3.71) stepping periods. Associations were not observed in SAs. Ethnic differences in associations between ambulation and physical function may exist in older adults which warrant further investigationi.

## Introduction

Research has indicated that the rates of physical frailty in adults over 70 years of age are as high as 20% O'Caoimh et al., 2021. Physical frailty is characterised by impaired physical functioning and difficulty in undertaking activities of daily living (Morley et al., 2013). It is clear from previous research that limitations to physical function can predict risk of disability, use of health care systems, admission to care homes, and mortality (Studenski et al., 2011). Modelling estimates suggest that physical frailty could increase healthcare costs by over 100% (Hajek et al., 2018).

Certain ethnic minority groups in economically developed Western countries are more likely to exhibit physical frailty than White ethnic groups and are more likely to start presenting with impairments to physical function at a younger age (Majid et al., 2020). South Asian (SA) people have specifically been reported to have lower levels of cardiovascular fitness, lower grip strength, and to score lower in physical function or walking assessments, than White European (WE) people (Ghouri et al., 2013; Ntuk et al., 2017). These observed differences in markers of fitness and function are clinically important as cardiorespiratory fitness and walking pace are strong markers of health status and longevity, and as such have been acknowledged as important cardiovascular risk factors (Kodama et al., 2009; Yates et al., 2017; Zaccardi et al., 2019). Whilst differences between ethnicities in the performance of laboratory walking and fitness tests have been established, it is unclear how these differences translate into habitual walking behaviours, movement intensity, and the impact of these on physical function.

Research has suggested that ≥100 steps/min is indicative of moderate-to-vigorous intensity physical activity (MVPA) and the number of steps above this threshold has been shown to be inversely associated with age, body mass index (BMI), the likelihood of presence of one or more diseases, higher levels of obesity, and poorer physical function (Dohrn et al., 2020; Tudor-Locke et al., 2018). Further, accumulating steps ≥100 steps/min has been recommended for reducing the risk of developing type 2 diabetes (T2D) (Tudor-Locke et al., 2017).

In addition, peak step cadence over a set time period may also be important for health. This can be defined as the highest number of steps/min in a continuous (i.e., peak 1 min) or non-continuous time period (i.e., peak 30 min) (Tudor-Locke et al., 2018). Higher peak 30-min step cadence has been shown to be inversely associated with cardiometabolic risk (Adams et al., 2019), with cadence values typically reducing with advancing age and increasing levels of obesity (Tudor-Locke et al., 2012).

However, whether these step cadence metrics are associated with physical function is unknown. This is an important limitation, particularly given that obesity is known to exacerbate the age-related decline in physical function. Moreover, previous research has typically been undertaken in WE populations, with a lack of research investigating whether associations differ across different ethnic groups. Given the accelerated decline in physical function observed in SAs, identifying metrics that represent behavioural patterns of ambulatory activity in free-living contexts, whilst having clinical and practical value, is crucial to inform future interventions. This is important as people with obesity typically experience impairments to physical function and decreased capacity to perform activities of daily living (Stoever et al., 2018); and considerable prevalence of impaired physical function has been observed in elderly obese individuals (Gretebeck et al., 2017). These metrics have also been shown to have negative relationships with several other cardiometabolic outcomes.

Therefore, this study aimed to quantify the associations between different step cadence metrics and physical function in healthy SA and WE older adults.

## Materials and methods

### Design and procedure

The analysis included data from the “Sedentary behaviour in older adults: investigating a new therapeutic paradigm (STAND UP) study” which recruited participants aged ≥60 years, free of chronic disease, in Leicester and Glasgow, UK between 2015 and 2017 (Yates et al., 2020). Physical function was measured using the 60-s Sit-to-Stand (STS-60) test. Free-living sitting, standing, and stepping were measured using the activPAL3™ accelerometer for 7 days on the thigh. Demographic information (sex, age, ethnicity, BMI) was collected via self- and assessor-administered questionnaires.

### Participants

STAND UP was a multi-centre study (Leicester and Glasgow) conducted across two work packages. The first (Leicester only) consisted of a cross-sectional study collecting accelerometer data during free-living conditions followed by a lab-based assessment of different physical activities under direct observation with the aim of developing age-appropriate cut-points for sedentary behaviour and MVPA in older adults within the UK. The second (Leicester and Glasgow) was a randomised crossover acute lab-based design aiming to investigate whether breaking sitting time with regular bouts of standing or light ambulation results in reduced area under the insulin curve in adults (Yates et al., 2020). Recruitment and measurements across both phases and sites were standardised to the same protocol. Participants were recruited through direct and opportunistic marketing using a variety of communication methods including mixed-media advertising (e.g., electronic, newspapers, newsletters, etc.), attending community events, and strategic placement and distribution of recruitment posters and promotional materials. Additionally, participants who had previously participated in research studies that had consented to being contacted about future research were sent information packs.

Participants were initially screened to confirm that they were ≥60 years of age, were able to walk without assistance from devices or other persons, were able to communicate in verbal and written English, were free from any condition or limitation that would render them unable to participate in the study, and able to give informed consent. Ethics approval was granted by East Midlands – Derby Research Ethics Committee (14/EM/1217). Participants provided written informed consent.

### Device-assessed physical activity and sedentary behaviour

Participants were asked to wear the activPAL3™ device (PAL Technologies, Ltd., Glasgow, UK) 24 h/day for 7 days on the midline anterior aspect of the right thigh. The initialisation, download, and extraction processes have been detailed elsewhere (Yates et al., 2020). For the purposes of this analysis, a valid waking day was defined as a day with <95% of time spent in any one behaviour (e.g., standing or sitting), ≥500 step events (1000 steps) and ≥10 h of waking hours data (Winkler et al., 2016). Participants were required to have at least 3 valid days of data to be included in the analysis (Kang et al., 2016). Output variables of interest included: waking wear time; time spent in postures of sitting, standing, and stepping; daily steps; and daily steps at different thresholds of step cadence, categorised as brisk walking (≥100 steps/min) or slow walking (<100 steps/min) (Tudor-Locke et al., 2018). The number of steps accumulated in each walking category was derived and the average per day calculated. In order to further explore the associations of stepping intensity, two outputs were derived. (1) Total number of slow and brisk steps undertaken per day, and (2) number of brisk steps undertaken in bouts of walking activity lasting at least 1 min per day. Slow steps were bounded at a lower rate of 50 steps/min and brisk steps were bounded at an upper rate of 150 steps/min in order to avoid very slow or fast frequencies of stepping that are unlikely to represent purposeful stepping.

The following mean peak step cadence variables were also created in STATA by using the event files and then matching the valid waking wear times identified from Processing PAL (code available on request): the mean step cadence for the most active 1 min, 30 min and 60-min periods. Briefly, the code generates these step cadence variables by: (1) assigning a cadence (step/event interval*60*2) to each individual step taken; (2) step cadence for each individual step is sorted by ascending order; (3) time intervals (not continuous) are collated in accordance with the time period of interest (1, 30, 60 min); (4) the average step cadence in the time period is identified as the mean. Frequently used techniques report peak cadence based on the accumulation of steps over a pre-defined epoch or across a walking event. This has the effect of diluting the true peak by averaging across the epoch or the duration of the event and has been criticised for actually measuring step accumulation as opposed to step cadence. In contrast, by assigning a step cadence to each individual step, we ensure the accurate capture of peak cadences (Granat et al., 2015; Stansfield, Clarke et al., 2015).

### Descriptive data

Participants were asked to report their date of birth, sex (male or female), ethnicity (participants self-identified to standard census definitions), smoking status, postcode, medical history, and current medications. Two ethnicity groups were created for this analysis, with White British, White Irish, or any other White background being grouped as WE and Indian, Pakistani, Bangladeshi, or any other South Asian background as SA (Yates et al., 2020).

Body weight and body composition (Tanita SC-330ST, Tanita, West Drayton, UK), height (Height Measure, Seca, Birmingham, UK), and waist circumference (midpoint between the lower costal margin and iliac crest) were measured to the nearest 0.1 kg, 0.1%, and 0.5 cm, respectively. Arterial blood pressure was measured in the sitting position (Omron Healthcare, Henfield, UK); three measurements were obtained and the average of the last two used.

### Physical function

Physical function was measured using the STS-60. The STS-60 has been described elsewhere (Gurses et al., 2018), Briefly, the test is performed on a chair of standard height (~45 cm) without arm rests. Participants are instructed to keep their arms stationary by placing hands on their hips. On the command “begin”, participants proceed to stand up and sit back down again as many times as they can within a 60-s period. Participants perform the movements at a self-selected pace and can stop at any time they wish. The number of complete sit-to-stand transitions in a 60-s period is recorded. The STS-60 is considered to be an effective measure of functional exercise performance and is well correlated with other measures of physical function such as the 6-min walk test (Reychler et al., 2018). Previous analysis has also shown the STS-60 to have “excellent” reliability (ICC = 0.927) and offers comparable results to the intermittent shuttle walk test (ISWT), estimated 1-repetition maximum for quadriceps strength, and cardiopulmonary exercise testing (Wilkinson et al., 2019).

### Statistical analysis

Descriptive variables are presented as numbers and percentages for each ethnic group (WE and SA). Descriptive statistics are reported as mean ± standard deviation (SD). Independent samples *t*-tests were conducted to compare differences between WEs and SAs in descriptive categories.

Differences between ethnic groups in the outcome of interest, STS60, were explored using generalised linear models with a Poisson distribution with an identity link. Models were adjusted for (1) age, sex, stature (height and weight), and fat-free mass and (2) model 1 plus brisk stepping and slow stepping.

Generalised linear models were used to assess whether slow and brisk stepping were associated with physical function. Models were stratified by ethnicity and adjusted for age, sex, stature (height and weight), and activPAL valid waking wear time. To account for the confounding effect of physical activity, models were also adjusted for overall physical activity category (<7500 or ≥7500 steps/day, based on previous estimates of steps/day in relation to physical activity recommendations (Tudor-Locke et al., 2011)). In addition, models were mutually adjusted whereby slow steps were adjusted for brisk steps and vice versa, to ensure one was not confounding the other. All data was analysed using a Poisson distribution with an identity link. All models were checked for multi-collinearity by examining the relationships between independent variables in the fully adjusted models. Models were initially run on total brisk and slow steps per day as the primary outcome and repeated for brisk steps per day undertaken in at least 1-min bouts. In order to further explore the association with brisk walking, the percentage of overall steps undertaken per day at a brisk cadence was calculated (brisk steps/overall steps × 100) and associated with STS-60 repetitions. The same modelling structure was used to assess the association between the most active 1-, 30-, and 60-min step cadence metrics and STS-60 repetitions.

Interaction terms were explored on the full dataset to assess whether associations with slow or brisk walking were modified by ethnicity.

A sensitivity analysis was conducted to investigate whether the main effects and interactions were attenuated after adjusting for differences in fat-free mass, which was not included in the main model given the potential to act as a mediator between the stepping intensity and the physical functional.

For ease of interpretation, the results of the multiple linear regression analyses are presented as the unstandardised beta coefficients (95% CI) per 1000 steps, per decile of brisk steps as a percentage of overall steps, and per 10 steps/min for mean peak step cadence. All data were analysed using IBM SPSS Statistics (version 24.0). A *p*-value of <0.05 was considered statistically significant for the main effects and interactions.

## Results

From the cohort of 108 participants, 4 were excluded due to missing activPAL data or missing STS-60 scores. A total of 104 individuals (age = 72 ± 5; 54% male) were included in the analysis. Within the 104 individuals included, 71 were WE (age = 72 ± 5, 54% male) and 33 SA (age = 71 ± 5, 55% male). Participant characteristics are displayed in Table 1. Both groups spent similar time sitting (9.0 ± 1.8 vs 9.0 ± 1.5 hours/day for WEs and SAs, respectively) and stepping (1.8 ± 0.6 vs 1.8 ± 0.5 hours/day for WEs and SAs, respectively). However, compared to WEs, SAs had significantly lower levels of overall steps per day (8986 ± 3450 vs 7780 ± 2340 steps/day, *p* = 0.040) and less brisk steps per day (5515 ± 2866 vs 3723 ± 2083 steps/day, *p* = 0.001). Mean peak 30-min and 60-min step cadence values also differed by ethnicity, with greater cadences seen in WEs (30 min, 117.7 ± 10.3 vs 111.8 ± 9.7 (*p* = 0.009) and 60 min, 107.1 ± 11.0 vs 100.8 ± 10.6 (*p* = 0.009) steps/min for WEs and SAs, respectively).

**Table 1.** Characteristics of study participants

	Population (*N* = 104)	WE (*N* = 71)	SA (*N* = 33)	*p value*
Sex	53.8% male	53.5% male	54.5% male	0.923
Age (years)†	71.7 ± 5.1	72.0 ± 5.1	71.3 ± 5.1	0.526
Weight (kg)	75.1 ± 14.3	77.7 ± 14.3	69.5 ± 12.7	0.006*
Height (cm)	164.0 ± 9.0	165.7 ± 9.0	160.4 ± 8.0	0.004*
Body Fat (%)‡	33.0 ± 7.9	32.5 ± 8.2	34.1 ± 7.3	0.344
Fat free mass (kg)	48.0 ± 14.8	50.9 ± 13.5	41.5 ± 15.6	0.002*
BMI (kg/m^2^)	27.8 ± 4.4	28.2 ± 4.6	26.9 ± 3.9	0.156
Waking wear time (hours/day)	15.4 ± 1.0	15.3 ± 1.0	15.7 ± 1.0	0.150
Sitting time (hours/day)	9.0 ± 1.7	9.0 ± 1.8	9.0 ± 1.5	0.935
Standing time (hours/day)	4.7 ± 1.3	4.5 ± 1.4	5.0 ± 1.2	0.116
Stepping time (hours/day)	1.8 ± 0.6	1.8 ± 0.6	1.8 ± 0.5	0.463
**Accumulated Daily Steps**				
Total (steps/day)	8603 ± 3179	8986 ± 3450	7780 ± 2340	0.040*
Slow (steps/day)	3657 ± 1434	3472 ± 1441	4057 ± 1354	0.052
Brisk (steps/day)	4946 ± 2763	5515 ± 2866	3723 ± 2083	0.001*
Proportion brisk	0.55 ± 0.18	0.59 ± 0.15	0.45 ± 0.19	<0.001*
Brisk (1-min bouts) (steps/day)	2506 ± 2114	2842 ± 2230	1785 ± 1650	0.008*
**Peak Step Cadence**				
Mean 1-min (steps/min)‡	156.1 ± 9.6	157.0 ± 9.5	154.0 ± 9.6	0.153
Mean 30-min (steps/min)‡	115.9 ± 10.42	117.7 ± 10.3	111.8 ± 9.7	0.009*
Mean 60-min (steps/min)‡	105.2 ± 11.2	107.1 ± 11.0	100.8 ± 10.6	0.009*

* < 0.05

**† *N* = 103; ‡ *N* = 100**

**WE, White European; SA, South Asian; BMI, body mass index; STS-60, 60-s sit-to-stand test**

**Figure 1.**
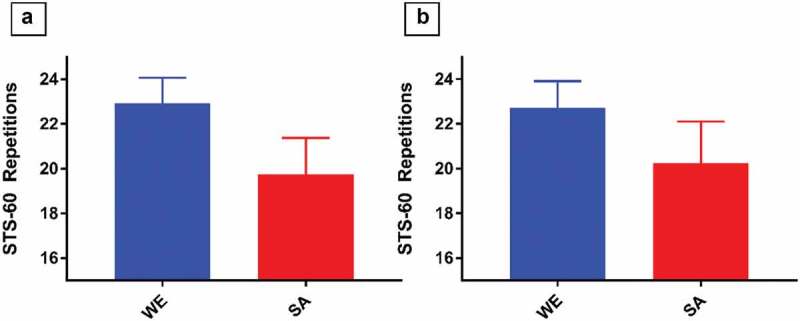
Estimated marginal mean STS-60 repetitions for White Europeans and South Asians Model 1 (panel 1A) adjusted for: age, sex, height, weight, and fat free mass. Model 2 (panel 1B) adjusted for: model 1 plus slow stepping and brisk stepping.

Compared to WEs, SAs scored lower in the STS-60 (23 (95% CI 21.77, 24.06) vs 20 (95% CI 18.13, 21.40) repetitions, *p* = 0.003; Figure 1). The difference was largely maintained after adjustment for slow and brisk stepping (*p* = 0.045), with a difference of 2.47 (0.06, 4.88) repetitions remaining between ethnicities (Table 2).

**Table 2.** Estimated marginal mean difference in STS-60 repetitions between White Europeans and South Asians

	WE EMM (95% CI)	SA EMM (95% CI)	Mean Difference (95% CI)	*p value*
Model 1	22.91 (21.77, 24.06)	19.77 (18.13, 21.40)	3.15 (1.09, 5.20)	0.003*
Model 2	22.71 (21.51, 23.91)	20.24 (18.38, 22.10)	2.47 (0.06, 4.88)	0.045*

* < 0.05

**Model 1 adjusted for: age, sex, height, weight, and fat free mass. Model 2 adjusted for: model 1 plus slow stepping and brisk stepping.**

**WE, White European; SA, South Asian; EMM, Estimated Marginal Mean; CI, Confidence Interval.**

**Figure 2.**
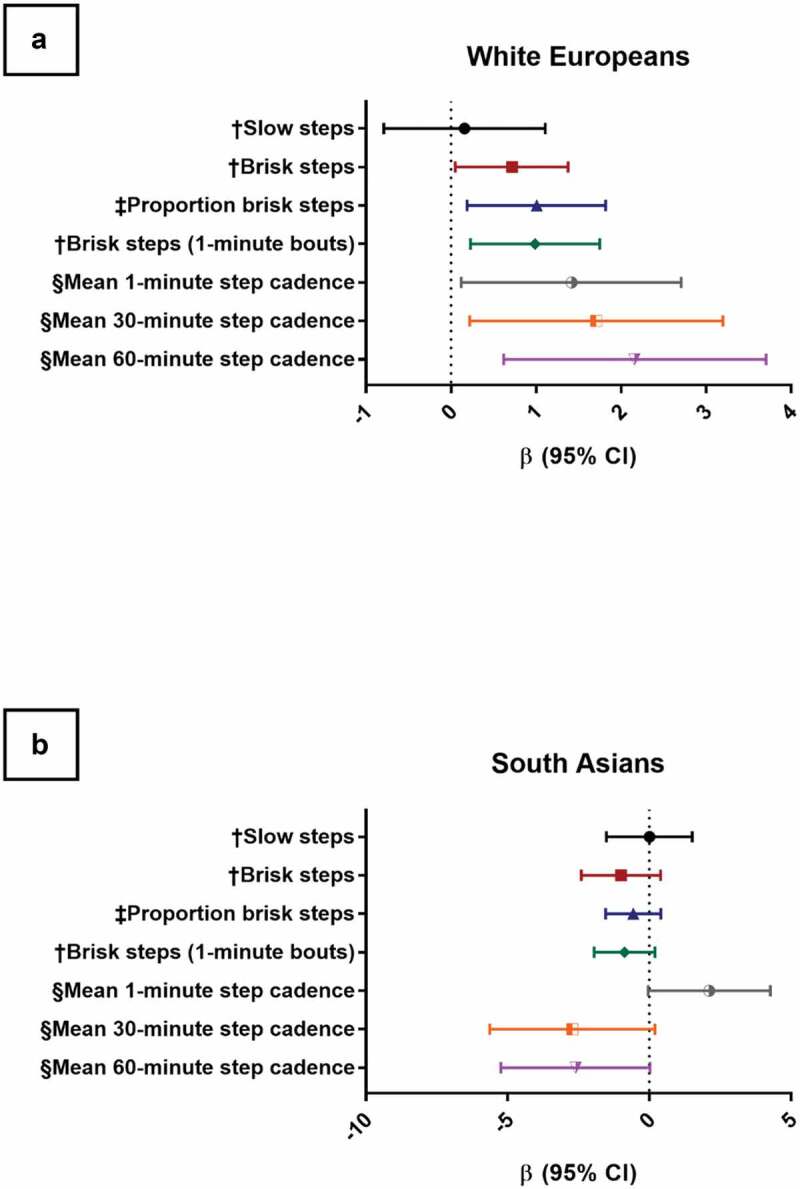
Forest plots of relationships between step cadence variables and STS-60 repetitions for White Europeans and South Asians. Model adjusted for: age, sex, height, weight, physical activity category, and accelerometer waking wear time. Slow/brisk steps mutually adjusted. Panel 2A represents relationships between directly measured step cadence and performance in the sit-to-stand-60 (STS-60) in White Europeans. Panel 2B represents relationships between directly measured step cadence and performance in the STS-60 in South Asians. † per 1000 steps, ‡ per decile, § per 10 steps/min.

The associations between measures of ambulation intensity and STS-60 are shown in Table 3 and Figure 2.

**Table 3.** Relationships between step cadence variables and physical function for White Europeans and South Asians

	WE (*N* = 71)		SA (*N* = 33)		*Interaction p value*
	*β (95% CI)*	*p*	*β (95% CI)*	*p*	
Slow steps†‡	0.16 (−0.79, 1.11)	0.747	0.01 (−1.51, 1.52)	0.994	0.645
Brisk steps†‡	0.72 (0.05, 1.38)	0.035*	−1.00 (−2.40, 0.40)	0.160	<0.001*
Proportion brisk steps§	1.01 (0.19, 1.82)	0.015*	−0.56 (−1.54, 0.41)	0.265	<0.001*
Brisk steps (1-min bouts)†‡	0.99 (0.23, 1.75)	0.010*	−0.87 (−1.95, 0.20)	0.112	<0.001*
Mean 1-min step cadence¶	1.42 (0.12, 2.71)	0.032*	2.12 (−0.04, 4.28)	0.054	0.377
Mean 30-min step cadence¶	1.71 (0.22, 3.20)	0.024*	−2.71 (−5.63, 0.20)	0.068	0.001*
Mean 60-min step cadence¶	2.16 (0.62, 3.71)	0.006*	−2.60 (−5.24, 0.03)	0.053	<0.001*

* < 0.05

**Model adjusted for: age, sex, height, weight, physical activity category, and accelerometer waking wear time.**

**† Mutually adjusted for the alternate (slow/brisk) metric.**

**‡ per 1000 steps, § per decile, ¶ per 10 steps/min.**

**WE, White European; SA, South Asian; CI, Confidence Interval.**

In WEs, the number of brisk steps and the proportion of total steps spent at brisk stepping were both associated with performance in the STS-60, with every 1000 brisk steps associated with 0.72 (95% CI 0.05, 1.38) more sit-to-stand repetitions and every 10% higher proportion of brisk steps taken compared to overall steps associated with 1.01 (95% CI 0.19, 1.82) more sit-stand repetitions. No associations were observed in SAs. The strength of association was significantly different to WEs (*p* < 0.01 for interaction). This pattern of association was similar in brisk steps accumulated in bouts of at least 1 min.

In WEs, all step cadence metrics for the most active 1, 30, and 60 min were associated with performance in the STS-60, with greater mean peak step cadences being associated with more STS-60 repetitions (mean 1-min *β* = 1.42 (95% CI 0.12, 2.71), mean 30-min *β* = 1.71 (95% CI 0.22, 3.20), and mean 60-min *β* = 2.16 (95% CI 0.62, 3.71)). No associations were observed in SAs, with the strength of association significantly different to WE for the 30-min and 60-min data (*p* < 0.01 for interaction).

Associations and interactions remained unchanged before mutual adjustment for slow and brisk steps and when further adjusting for fat-free mass (see Supplementary Tables 1 and 2, respectively).

## Discussion

This study sought to assess the associations between different step cadence metrics, describing habitual stepping intensity, and physical function assessed by the STS-60 in older adults, and whether these associations differed between SAs and WEs. The results demonstrated that in WEs, a greater number of brisk steps taken per day, a higher proportion of brisk steps taken per day, or higher peak stepping cadences were associated favourably with physical function. In SAs, levels of brisk walking and physical function were lower than in WEs and there was no association between these factors, regardless of how stepping intensity or cadence was assessed.

Recent analysis of large cohort studies concluded that although the higher intensity of peak 1-min and peak 30-min step cadence was associated with lower mortality rates, after adjustment to total steps per day, these associations were largely attenuated (Saint-Maurice et al., 2020). However, other research has demonstrated that gait speed can be an important factor in the development of impaired physical function in both extremely frail and more robust, largely white, populations (Judge et al., 1996). Some research has suggested that slower walking speeds are associated with disability, frailty, muscular weakness, falls, and poor performance in step cadence assessments (Pamoukdjian et al., 2015). In addition, various functional tasks (particularly those which are characteristic of sit-to-stand/stand-to-sit movements) have also been associated with step cadence in the previous research. In particular, hip extension, hip flexion, knee extension, and ankle plantarflexion have all been significantly associated with changes in step cadence (Lim et al., 2017).

However, these studies have assessed walking pace through laboratory tests. Although this is not the first study to assess the impact of objectively measured habitual stepping intensity and cadence on measures of physical function, existing studies have typically focussed on specific clinical populations and have not included formal analysis by ethnicity (Gothe & Bourbeau, 2020). Whilst habitual stepping intensity is strongly associated with physical function in WEs, there was no association in SAs with the magnitude of association significantly different from WEs. In addition, the lower levels of physical function in the SA individuals were independent of differences in brisk and slow stepping activity. Our cross-sectional findings are consistent with the wider literature. Whilst the effect of exercise on markers of cardio-metabolic health in SA populations has been positive (Albalawi et al., 2017), the effects on measures of fitness, function, and strength have been more equivocal. A systemic review of individuals with T2D identified two studies that assessed functional outcomes, one of which proposed differences compared to control and one of which did not (Albalawi et al., 2017). Furthermore, whilst exercise training has been shown to increase muscle strength in SA populations (Albalawi et al., 2017), there is evidence that adaptions to strength, in particular lower body strength, are slower and to a lower magnitude than WEs. In the analysis of the responses to a 6-week progressive resistance exercise training programme, WEs were found to have considerably greater responses to lower body muscular strength (Knox et al., 2017). However, other researchers have demonstrated that SAs respond robustly to resistance exercise, increasing muscle mass and function to a similar extent as WEs (Alkhayl, 2018). Taken together, these studies suggest that mechanisms other than lower levels of physical activity may be needed to explain underlying impairments or differences in muscle physiology, physical function, and fitness in SA populations. Genetic, epigenetic, and foetal programming are all possible candidates that have been identified previously and require future research (Sattar & Gill, 2015). The cultural context for why individuals engage in walking may also be important; for example, brisk waking for exercise may be more strongly linked to recreation and leisure in WE populations, whereas it may be less culturally appropriate for SA populations. Consequently, where brisk walking is undertaken in SA populations, it may be more likely to take place in non-leisure or non-recreational contexts. Differences in contexts may in turn influence associations with health (Horne & Tierney, 2012).

Another potential factor contributing to the comparatively low levels of physical function in SA participants could be the inherently lower levels of lean mass. Indeed, in the present study, body composition analysis revealed that the mean percentage of fat-free mass was ~5 centiles lower in the SAs than in the WEs. Body composition analyses in SA men and women from other studies have also consistently shown lower levels of lean mass in SAs than in WEs (Misra & Khurana, 2011). Previous research has also indicated significantly lower levels of muscular strength, muscular perfusion, and muscular oxidative capacity in SAs compared to WEs, which remained constant even after control for various cardiometabolic factors – including prevalence of T2D (Ntuk et al., 2017). However, in the present analysis, differences between ethnicities in STS-60 remained independent of fat-free mass, as did ethnic differences in the association between stepping intensities and STS-60.

A strength of this study is the use of the activPAL device to measure step cadence. The activPAL has previously been found to be highly accurate in determining step cadence at speeds ≥0.5 m/s (Stansfield et al., 2015). The study is potentially limited by physical function being assessed by only one measure, the STS-60 test. However, this test has been shown to have good measurement properties, is an established measure of overall functional ability, and has been associated with other measures of physical function – including walk tests, 1-repetition maximum testing, and cardiopulmonary exercise testing (Reychler et al., 2018; Wilkinson et al., 2019). The STAND UP cohort of ~100 older adults from two major cities with high densities of SAs and WEs, is a relatively small sample and consists of a small number of SAs compared to WEs. This may affect the power and precision of the effect estimates. The nature of the cohort, which only included healthy volunteers, may not be generalisable to a typical older adult population. Finally, as this study is observational in nature, results could be explained by unmeasured confounders and causality, including the direction of causality, cannot be tested.

In conclusion, these results highlight that, compared to WEs, SAs have lower levels of ambulatory activity, lower physical function, fewer steps taken at a brisk pace, and lower mean peak step cadence for a range of time thresholds. In WEs only, we demonstrated that brisk walking, but not slow walking, is associated with physical function. This may have important implications for future intervention design in this area. By continuing to explore this topic further, researchers will be better equipped to tailor interventions to appropriately address the health issues of different ethnic groups. It is recommended that the relationships between step cadence, ethnicity, and physical function be further explored in cohorts with chronic disease.

### Perspective

The results highlighting the importance of brisk stepping for WEs in this study are potentially clinically meaningful. For example, two repetitions have been reported as the minimum clinically meaningful difference in results from the STS-60 (Bohannon & Crouch, 2019). Based on the results of the present study, walking an additional 2777 brisk steps per day relates to a difference of two STS-60 repetitions. This equates to approximately 28 min of brisk walking per day, which is consistent with the minimum physical activity recommendations for health (Iliodromiti et al., 2016). Alternatively, a difference of 20% in the proportion of overall steps undertaken at a brisk cadence (e.g., moving from 50% to 70% of total steps at a brisk cadence) was also related to approximately two STS-60 repetitions, independent of overall physical activity levels. Finally, increasing the mean peak 1-min step cadence by 15 steps/min; mean peak 30-min step cadence by 12 steps/min; or mean peak 60-min step cadence by 9 steps/min are all associated with a difference of two STS-60 repetitions.
